# Repurposing screens reveal a role for PKCη and NF1 in SARS-CoV-2 infection

**DOI:** 10.1371/journal.ppat.1014417

**Published:** 2026-07-23

**Authors:** Jorge A. Acuña, Jesse Miller, Smita Bhutda, Kasirajan Ayyanathan, Brinda Kamalia, Kanupriya Whig, David Nguyen, Yongqing Zhu, Benoît Laleu, Timothy Wells, Kirandeep Samby, Andrew Pekosz, David C. Schultz, Sara Cherry

**Affiliations:** 1 Department of Pathology and Laboratory Medicine, Perelman School of Medicine, University of Pennsylvania, Philadelphia, Pennsylvania, United States of America; 2 Department of Biochemistry and Biophysics, Perelman School of Medicine, University of Pennsylvania, Philadelphia, Pennsylvania, United States of America; 3 MMV Medicines for Malaria Venture, ICC International Center Cointrin, Geneva, Switzerland; 4 W. Harry Feinstone Department of Molecular Microbiology and Immunology; Johns Hopkins Bloomberg School of Public Health, Baltimore, Maryland, United States of America; Duke-NUS Medical School, SINGAPORE

## Abstract

SARS-CoV-2 continues to circulate with the emergence of variants that evade existing immunity. However, all strains rely on conserved host factors for entry, making them attractive targets for host-directed antivirals. SARS-CoV-2 engages the ACE2 receptor and can enter cells through two alternative pathways depending on cell type: Spike cleavage at the plasma membrane by TMPRSS2, or within endocytic compartments by cathepsins. Cleavage triggers Spike-mediated membrane fusion and release of the viral genome. To discover small-molecule inhibitors of entry, we first screened compounds against live virus and active candidates were then tested using recombinant VSV expressing SARS-CoV-2 Spike, with VSV expressing its native glycoprotein serving as a control. This approach identified known and novel TMPRSS2 inhibitors, as well as Staurosporine and Retro-2.1. Both compounds inhibited infection in both TMPRSS2-dependent and –independent cell types. Entry bypass studies revealed that Staurosporine acts upstream of Spike cleavage, while Retro-2.1 functions downstream. Mechanistic studies in Calu-3 cells showed that Staurosporine, a pan-PKC inhibitor, blocks entry via PKCη, a pro-viral factor acting before Spike cleavage. Retro-2.1 targets NF1, which promotes infection downstream of Spike cleavage. Together, our screening pipeline identified inhibitors that block SARS-CoV-2 entry at distinct stages and revealed host factors that may inform the development of current and novel antiviral strategies.

## Author summary

SARS-CoV-2, the virus responsible for the COVID-19 pandemic, continues to spread and evolve into new variants capable of evading existing immunity. However, all variants rely on conserved human cell factors to successfully enter and infect diverse cells. Identifying host factors required for infection may reveal additional conserved targets for novel antivirals that work broadly against different variants. We used a two-step screening approach to discover viral entry inhibitors, uncovering both known and new inhibitors, including Staurosporine and Retro-2.1, which blocked infection across cell types. Mechanistically, we found that the pan-PKC inhibitor, Staurosporine, interferes with an early step in the viral entry process controlled by the host factor PKCη in Calu-3 cells. Additionally, we found that the retrograde trafficking inhibitor, Retro-2.1, blocks a later step that depends on the host factor, NF1 in Calu-3 cells. Overall, our screening pipeline identified inhibitors that block SARS-CoV-2 entry at distinct stages and revealed host factors that may inform the development of current and novel antiviral strategies.

## Introduction

The *Coronaviridae* family is comprised of positive-sense RNA viruses which possess zoonotic capabilities with diverse pathogenicity [1,2]. Whereas seasonal human coronaviruses cause mild respiratory symptoms, severe acute respiratory syndrome coronavirus (SARS-CoV) and Middle East respiratory syndrome coronavirus (MERS-CoV) cause severe disease [3,4]. Most recently, SARS-CoV-2 emerged in 2019, causing a range of mild respiratory complications to severe illness including difficulty breathing, pneumonia, and death. While vaccines and therapeutics have mitigated disease progression, concerns remain over emerging variants with characteristics of increased transmissibility, immune evasion properties, or pathogenesis, namely, Variants of Concern (VoC) that continue to evolve [5]. The evolution of these VoCs involves numerous substitutions of the viral Spike glycoprotein which allow new strains to evade neutralizing antibodies and have been implicated in contributing to the pathogenesis of these strains [5,6]. The evolved Spike proteins can thus re-infect individuals possessing neutralizing antibodies to previous strains and additional mutations throughout the virus may confer resistance to antivirals targeting other viral proteins [7–9]. While Spike has evolved to evade antibodies, it continues to use host factors including the angiotensin converting enzyme 2 (ACE2) receptor for entry. Therefore, host-directed antivirals against this conserved entry pathway may provide potential for broader protection with a reduced likelihood of developing resistance.

Viral entry is a critical step of infection and can be targeted by therapeutics [10,11]. The SARS-CoV-2 Spike glycoprotein consists of an S1 subunit that binds to the host cell receptor, ACE2, and an S2 subunit containing the S2’ cleavage site and fusion peptide [12,13]. Following binding to ACE2, the viral glycoprotein must be processed by cellular proteases to trigger conformational changes required for membrane fusion, and eventual release of the viral genome into the cytoplasm [14]. Two distinct proteases have been shown to process Spike in different contexts. The plasma-membrane associated serine protease 2 (TMPRSS2) processes Spike at the plasma membrane while cathepsin proteases can process Spike from within acidified endosomes contexts [15–17].

SARS-CoV-2 primarily infects the respiratory tract in humans. These cells express the ACE2 receptor and TMPRSS2 apically and thus TMPRSS2 is thought to be the major protease utilized in these cells [18,19]. In addition to the respiratory tract, ACE2 and TMPRSS2 are highly expressed apically in the intestinal epithelium [18–20]. In contrast, in many other cell types, ACE2 is expressed but not TMPRSS2 [18,21]. In those cells SARS-CoV-2 infection is dependent on endosomal uptake and processing by acid-dependent cathepsins [15–17]. To explore these different pathways, we utilize respiratory Calu-3 and intestinal Caco-2 cells which express ACE2 and TMPRSS2 [15,16,22]. We also utilize hepatocyte Huh7.5 which endogenously express ACE2 but not TMPRSS2 and respiratory A549 cells ectopically expressing ACE2, but not TMPRSS2 [15–17]. We confirm that in cells that express TMPRSS2, the plasma membrane entry pathway is preferentially utilized, while in cells that do not express TMPRSS2, cathepsins process Spike in endosomal compartments [15,16]. This is best exemplified using inhibitors: the TMPRSS2 inhibitor Camostat is active in Calu-3 and Caco-2 but not in other cell types while inhibitors of different steps in the endosomal pathway inhibit infection in Huh7.5 and A549-ACE2 cells but not in Calu-3 or Caco-2 cells [15,16]. Cathepsins are acidic proteases that require a low pH for their activity and thus inhibitors of either endocytosis (Apilimod inhibits PIKfyve required for trafficking of endosomes to lysosomes) or inhibitors of endosomal acidification (Hydroxychloroquine and ammonium chloride) or direct inhibitors of cathepsin enzymatic activity (Aloxistatin and SB412515) block SARS-CoV-2 entry in TMPRSS2-deficient cells [15,16].

Additional cellular factors including attachment factors can facilitate binding of SARS-CoV-2 to the plasma membrane and thus viral entry. For example, heparan sulfate and sialic acids bind to SARS-CoV-2 Spike for initial attachment and additional TMPRSS2 homologs (TMPRSS4, TMPRSS11D, and TMPRSS13) are also thought to facilitate surface processing [23–26]. It is thought that after ACE2 binding, once TMPRSS2 cleaves Spike, the virus fuses at the plasma membrane and additional entry factors are not required. In contrast, in cells lacking TMPRSS2, after virus binding to ACE2, endosomal uptake brings virions into acidified compartments for cathepsin cleavage and fusion at endosomal membranes. Other than ACE2, there are no known commonalities in the two entry pathways. Therefore, we suggest that identifying small molecules capable of blocking both entry routes may provide insight into mechanisms used downstream of Spike cleavage and facilitate development of a new class of antivirals.

To identify additional entry requirements, we screened for small molecule inhibitors of SARS-CoV-2 entry. First, we screened for inhibitors of viral infection using wild type SARS-CoV-2 in Calu-3 cells followed by a second screen for those with a specific impact on entry. We validated 112 candidates as inhibitors of SARS-CoV-2 infection. Next, we screened these 112 compounds for activity against recombinant vesicular stomatitis virus (VSV) encoding Spike (VSV-S) versus VSV encoding the endogenous glycoprotein G (VSV-G) [27,28]. From this second screen we identified two canonical (Camostat and Nafamostat) and two non-canonical TMPRSS2 inhibitors (Avoralstat and UK-371804) along with two compounds (Staurosporine and Retro-2.1) with previously unknown roles in SARS-CoV-2 entry.

We found that Staurosporine and Retro-2.1 inhibit SARS-CoV-2 in both TMPRSS2-dependent and TMPRSS2-independent cell lines, and do not impact ACE2, suggesting they target a new step in the entry pathway. Using functional binding and trypsin bypass assays, we found that these inhibitors target distinct steps: Staurosporine blocks entry at or prior to TMPRSS2 engagement whereas Retro-2.1 blocks entry post-TMPRSS2 engagement. We next set out to identify the host targets of these inhibitors.

Staurosporine is a well-established inhibitor of protein kinase C (PKC). Protein kinase C (PKC) consists of a family of serine/threonine protein kinases involved in signaling pathways controlling cell growth, proliferation, differentiation, and cell death [29]. Moreover, PKC signaling has been shown to regulate receptor desensitization and internalization, membrane trafficking, and endocytosis [30–34]. There are nine mammalian PKC genes with additional forms resulting from splicing [29,35]. Different isoforms can be activated by phorbol esters (plant derivatives with tumor-promoting properties) and are classified into one of three subgroups depending on their requirements for activation [29,36]. Conventional (c)PKCs consist of the α, βI, βII, and γ isoforms and require calcium (Ca^2+^) and diacylglycerol (DAG) for activation. Novel (n)PKCs consist of the δ, ε, η, and θ isoforms and require DAG but not Ca^2+^ for activation. Atypical (a)PKCs consist of ζ and ι/λ and rely on phospholipids and protein-protein interactions for activation [37]. PKC isoform expression is cell-type dependent and activation and duration can be stimuli-specific [29,35,37]. Thus, target substrates and subsequent cellular responses within a cell type are determined by the stimulus and array of PKCs activated.

By screening a panel of isozymes we identified PKCη as proviral for SARS-CoV-2 entry upstream of TMPRSS2 cleavage. Further, we found that Staurosporine treatment reduced expression of PKCη. Retro-2.1 has been shown to bind SEC16A, MAP3K5, and NF1. Therefore, we screened the three known cellular binding partners of Retro-2.1 in Calu-3 cells and identified NF1 as proviral for SARS-CoV-2 infection. As we found for Retro-2.1, NF1 is required downstream of TMPRSS2 engagement. Altogether, our findings establish Staurosporine and Retro-2.1 as inhibitors of SARS-CoV-2 entry and identify PKCη and NF1 as proviral host factors for SARS-CoV-2 infection.

## Results

### Two tier screening identifies inhibitors of SARS-CoV-2 entry

**Fig 1:**
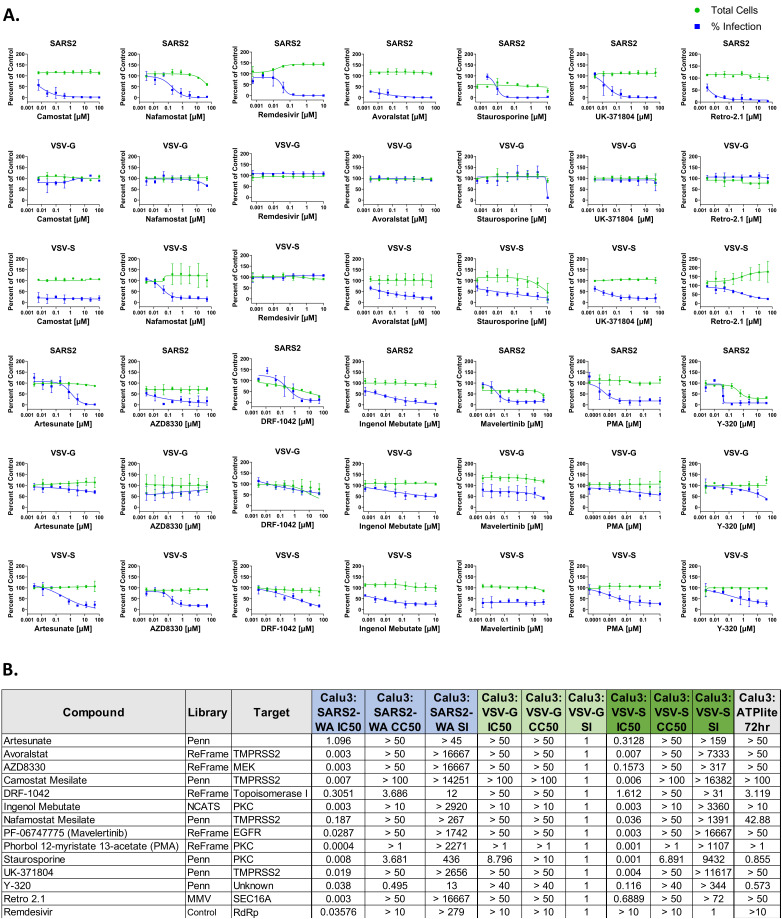
Identification of SARS-CoV-2 Entry Inhibitors. **(A)** Calu-3 cells were pre-treated with the indicated SARS-CoV-2 antivirals in 8-point dose-response and infected with SARS-CoV-2 (48 hours; MOI 0.5), VSV-G (24 hours; MOI 0.05), and VSV-S (24 hours; MOI 5) to achieve similar levels of infection (20-50% of control). Cells were processed for automated microscopy and image analysis quantifying total cell numbers (green) (nuclei+) and percent infection (blue) (Spike+ or dsRNA + /nuclei+) or (GFP + /nuclei+) to determine Percent of Control (POC) % Positive. Compounds included the 10 validated SARS-CoV-2 antivirals from the Pandemic Response Box and the 102 previously validated SARS-CoV-2 antivirals from the PENN, NCATS, and ReFrame repurposing libraries. Data are presented as mean values of n = 2 independent biological replicates ± SD. **(B)** Screening 112 compounds identified 13 with an SI > 10 for SARS-CoV-2 and VSV-S and an SI = 1 for VSV-G. Each compound’s IC50, CC50, and SI is listed for each virus along with ATPlite cytotoxicity results following 72-hour treatments.

We previously screened repurposing libraries from PENN, NCATS, and ReFrame for small molecules active against SARS-CoV-2 in human respiratory Calu-3 cells and identified 102 compounds with antiviral activity against SARS-CoV-2 with a Selective Index (SI = CC_50_/IC_50_)>10 [38]. We also screened the Pandemic Response Box assembled by Medicines for Malaria Venture (MMV) and the Drugs for Neglected Disease Initiative (DNDi) (400 compounds) using the same conditions and identified 16 compounds that reduce SARS-CoV-2 infection below 40% while maintaining cell viability above 80% in replicate screens (S1A-S1B Fig, S1 Table**)** [16,38,39]. Dose-response studies, quantifying the percent infection, and cytotoxicity, identified 10 compounds with an SI > 10 against SARS-CoV-2 in Calu-3 cells (S2 Table). Among these 112 validated candidates are the canonical TMPRSS2 entry inhibitors Camostat and Nafamostat. Therefore, we sought to identify additional compounds within this set that may block SARS-CoV-2 entry. To identify drugs that specifically block entry we took advantage of recombinant VSV expressing the endogenous glycoprotein G (VSV-G) and VSV expressing SARS-CoV-2 Spike (VSV-S) [27,28]. We compared the activity of the 112 compounds to identify those that selectively block infection of VSV-S compared to VSV-G. We and others have found that in cells that express TMPRSS2, TMPRSS2 inhibitors are active while endosomal inhibitors are not [15,16]. Indeed, Camostat and Nafamostat inhibit SARS-CoV-2 and VSV-S, but not VSV-G in Calu-3, demonstrating the utility of the VSV assay in identifying TMPRSS2-dependent entry inhibitors (Fig 1A-1B**)**. Likewise, in these cells, the endosomal inhibitors Aloxistatin, Hydroxychloroquine, and SB-412515 are not active against SARS-CoV-2 or VSV-S (S2A-**S2B Fig**). As a control to distinguish drugs that selectively block SARS-CoV-2 downstream of entry we used Remdesivir, a nucleoside analog targeting the RNA-dependent RNA polymerase (RdRp), which blocks SARS-CoV-2 RNA replication but not VSV-S nor VSV-G **(**Fig 1A-1B). After testing the 112 compounds, we identified 13 compounds with an SI > 10 for VSV-S and SI = 1 for VSV-G **(**Fig 1A-1B, S2 Table**)**. We further explored these candidates and deprioritized AZD8330 (MEK inhibitor), Mavelertinib (EGFR inhibitor), and DRF-1042 (Topoisomerase inhibitor) because additional MEK, EGFR, and Topoisomerase inhibitors had no selectivity against VSV-S suggesting off-target effects (S1 Table). Artesunate was deprioritized as its activity against SARS-CoV-2 was previously described [40]. Ingenol Mebutate, PMA, and Y-320 were also deprioritized due to their modest activity against VSV-G at higher concentrations (Fig 1A-1B). Thus, we focused on four compounds (Avoralstat, Staurosporine, UK-371804, and Retro-2.1) that inhibited VSV-S in a dose-dependent manner without inhibiting VSV-G (Fig 1A-1B).

### Staurosporine and Retro-2.1 block infection in both TMPRSS2-dependent and -independent cell types

**Fig 2:**
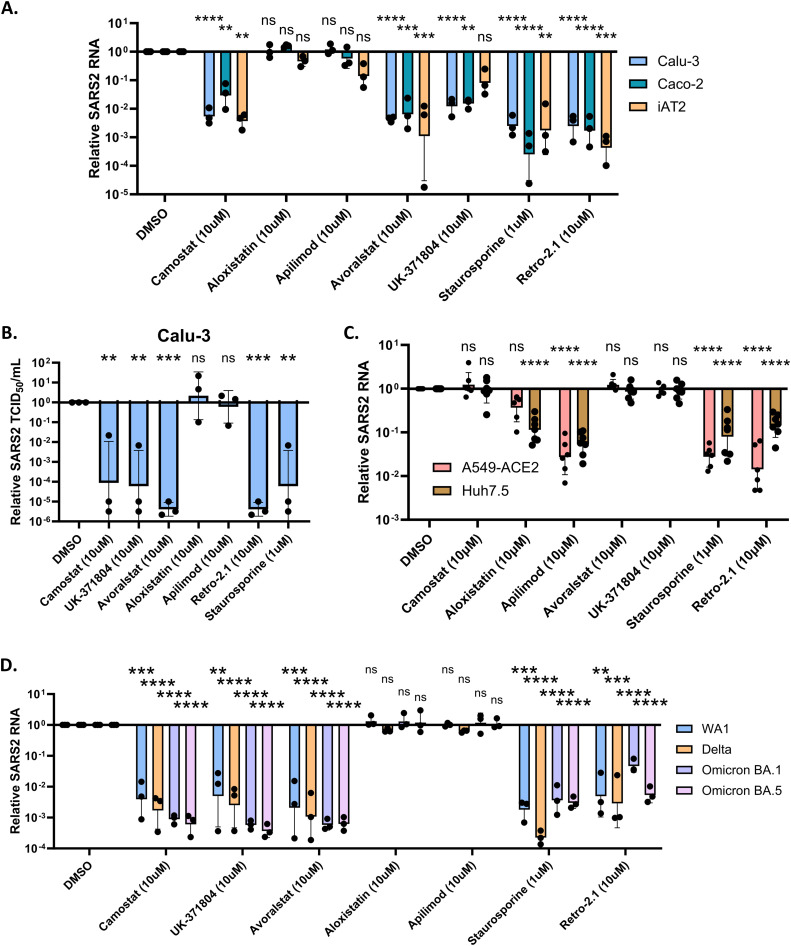
Staurosporine and Retro-2.1 Block Entry in Both TMPRSS2-dependent and –independent Cell Types. **(A)** Calu-3, Caco-2, and iAT2 cells were pre-treated with vehicle (DMSO) or the indicated compounds at the indicated concentrations and infected with SARS-CoV-2 (MOI 0.2) for 48 hours prior to processing for RT-qPCR analysis of viral RNA (Nucleocapsid). **(B)** Calu-3 cells were pre-treated with vehicle (DMSO) or the indicated compounds at the indicated concentrations and infected with SARS-CoV-2 (MOI 0.2) for 48 hours prior to supernatant collection. VeroTMPRSS2 cells were infected with serial dilutions of supernatants for 48 hours to determine viral titers by TCID_50_. **(C)** A549-ACE2 and Huh7.5 cells were pre-treated with vehicle (DMSO) or the indicated compounds at the indicated concentrations and infected with SARS-CoV-2 (MOI 0.2) for 24 hours prior to processing for RT-qPCR analysis of viral RNA (Nucleocapsid). **(D)** Calu-3 cells were pre-treated with vehicle (DMSO) or the indicated compounds at the indicated concentrations and infected with SARS-CoV-2 ancestral WA1, Delta, Omicron BA.1, and Omicron BA.5 variants (MOI 0.2) for 48 hours prior to processing for RT-qPCR analysis of viral RNA (Nucleocapsid). Means + SD with individual biological replicates are shown. Significance for relative viral RNA and titers were calculated using One Way ANOVA with Dunnett Correction on control conditions (*p < 0.05, **p < 0.01, ***p < 0.001, ****p < 0.0001; ns, no significance).

SARS-CoV-2 entry is dependent on either TMPRSS2 or endosomal cathepsins depending on the cell type [15,16]. Therefore, we tested the activity of our candidates in a panel of cell lines that utilize these two distinct entry pathways. First, we used an orthogonal assay to determine the magnitude of inhibition quantifying viral RNA using RT-qPCR in Calu-3 cells. We included Camostat as a positive control to inhibit TMPRSS2-dependent entry and Aloxistatin and Apilimod as inhibitors of the endosomal entry pathway (Fig 2A) [16]. SARS-CoV-2 viral replication was inhibited by Camostat as well as our four identified compounds, but not by Aloxistatin or Apilimod as measured by RT-qPCR (Fig 2A). Further, we confirmed that these inhibitors reduced viral titers in Calu-3 cells (Fig 2B). We thus tested two additional cell types that are TMPRSS2 dependent: Caco-2 cells which are intestinal epithelial cells expressing endogenous ACE2 and TMPRSS2 and human induced pluripotent stem cell (iPSC)-derived alveolar type II cells (iAT2) which express endogenous ACE2 and TMPRSS2 and are a more physiologically relevant cell model [15,16,20,41,42]. Consistent with the results in Calu-3 cells, SARS-CoV-2 viral replication was inhibited by Camostat as well as our four newly identified compounds (Fig 2A). Endosomal inhibitors failed to inhibit infection in both Caco-2 and iAT2 cells **(**Figs 2A **and** S2C-S2E**)**.

To determine whether these compounds were specific to TMPRSS2-dependent entry, we examined their activity in cell lines expressing ACE2, but devoid of TMPRSS2 including A549 cells ectopically expressing ACE2, and Huh7.5 cells which express ACE2 endogenously but are deficient in TMPRSS2 expression [15–17]. As expected, the endosomal inhibitor Apilimod, and the cathepsin inhibitor Aloxistatin blocked infection in A549-ACE2 and Huh7.5 cells, demonstrating endosomal entry in these cells (Fig 2C). Moreover, the TMPRSS2 inhibitor Camostat did not block SARS-CoV-2 infection in A549-ACE2 or Huh7.5 cells (Figs 2C and S2C-S2E). Staurosporine and Retro-2.1, but not Avoralstat and UK-371804, blocked infection in both cell types (Fig 2C). Avoralstat and UK-371804 are serine protease inhibitors and recent studies found they are also enzymatic inhibitors of TMPRSS2, and our data support this activity [43,44]. Staurosporine is a natural product and a potent inhibitor of protein kinases, in particular the PKC family of kinases [45,46]. Retro-2.1 is a small molecule derivative Retro-2, both of which inhibit retrograde trafficking [47,48].

### Staurosporine and Retro-2.1 block infection across SARS-CoV-2 variants

SARS-CoV-2 variants, particularly the Omicron lineage, encode a large number of changes in the Spike protein that can impact which entry pathway is utilized [49,50]. Thus, we tested the activity of our candidate compounds against SARS-CoV-2 variants including Alpha, Beta, Delta, Omicron BA.1, and Omicron BA.5. In Calu-3 cells, Camostat treatment or a combination of Camostat and Aloxistatin, but not Aloxistatin alone, inhibited infection of all variants, suggesting that these variants use a TMPRSS2-dependent entry mechanism in these cells (S3A Fig). We then treated TMPRSS2-deficient A549-ACE2 and Huh7.5 cells with Camostat, Aloxistatin, or a Camostat/Aloxistatin combination prior to infection with either the ancestral WA1 or Omicron BA.1 strains. Aloxistatin and the Camostat/Aloxistatin treatments, but not Camostat alone, inhibited infection of both variants, suggesting that endosomal TMPRSS2-independent entry is required for both strains in these cells (S3B Fig). After confirming that SARS-CoV-2 variants have similar sensitivity to the known entry inhibitors, we tested Avoralstat, UK-371804, Staurosporine, and Retro-2.1 activity in Calu-3 cells and found that each compound blocked infection of all variants (Figs 2D and S4A-S4B**)**. Given that both Staurosporine and Retro-2.1 blocked infection independent of TMPRSS2 expression and across variants, this suggests they may target a common step between the two entry pathways.

### TMPRSS2 inhibitors and Staurosporine but not Retro-2.1 impact MERS-CoV infection

**Fig 3:**
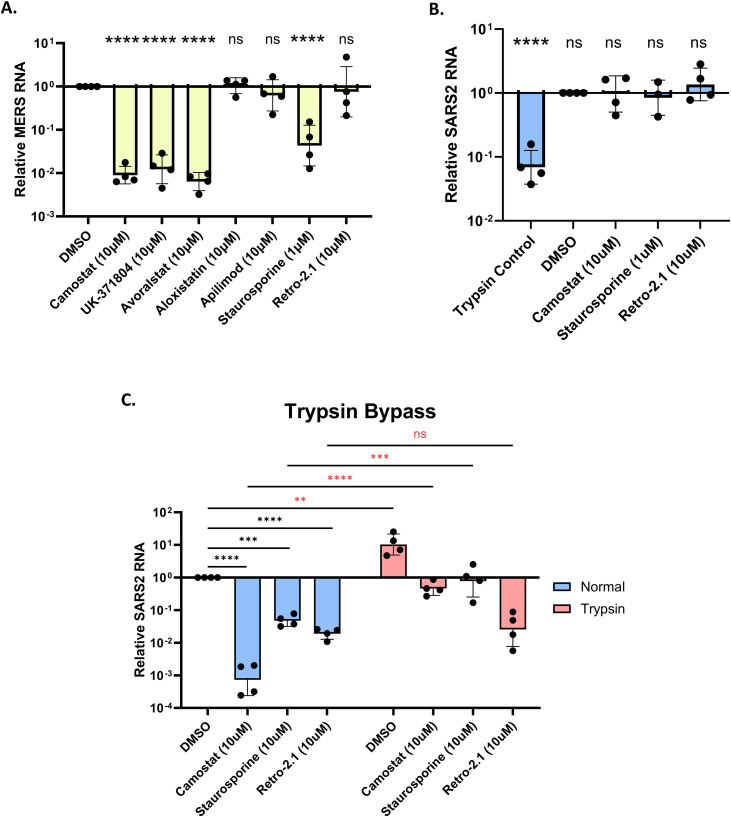
Staurosporine and Retro-2.1 Inhibit Entry at Distinct Steps. **(A)** Calu-3 cells were pre-treated with vehicle (DMSO) or the indicated compounds at the indicated concentrations and infected with MERS-CoV (MOI 0.2) for 24 hours. Cell lysates were processed for RT-qPCR analysis of viral infection (Nucleocapsid RNA). **(B)** Calu-3 cells were pre-treated with vehicle (DMSO), the indicated compounds at the indicated concentrations, or left untreated and infected with SARS-CoV-2 (MOI 2) at 4°C for one hour prior to washing with PBS to remove unbound virus. 0.25% trypsin was added to untreated cells with SARS-CoV-2 and incubated at 37°C for 5-10 minutes to dissociate cells and bound virus as a positive control. Cell lysates were processed for RT-qPCR analysis of viral infection (Nucleocapsid RNA). **(C)** Calu-3 cells were pre-treated with vehicle (DMSO) or the indicated compounds at the indicated concentrations and infected with SARS-CoV-2 (MOI 2) at 4°C for 30 minutes in duplicate. Cells were washed with PBS to remove unbound virus and either fresh media or 10µg/mL of TPCK-treated trypsin was added and incubated for five minutes at 37°C. Cells were then washed and replaced with fresh media and compounds for 20 hours. Cell lysates were processed for RT-qPCR analysis of viral infection (Nucleocapsid RNA). Means + SD for individual biological replicates are shown. Significance for relative viral RNA was calculated using One Way ANOVA with Dunnett Correction on DMSO control and significance for relative viral RNA of the trypsin bypass was calculated using One Way ANOVA with Sidak Correction for multiple comparisons (*p < 0.05, **p < 0.01, ***p < 0.001, ****p < 0.0001; ns, no significance.

We then tested the activity of these compounds against the distantly related coronavirus, MERS-CoV. MERS-CoV utilizes dipeptidyl peptidase 4 (DPP4) as a receptor for cellular binding, and not ACE2; however, MERS does depend on TMPRSS2 for entry [51]. As expected, treatment with the TMPRSS2 inhibitors Camostat, UK-371804, and Avoralstat blocked MERS-CoV in Calu-3 cells, whereas treatment with the endosomal inhibitors Aloxistatin and Apilimod had no effect, confirming the use of TMPRSS2-dependent entry (Fig 3A). Next, we tested Staurosporine and Retro-2.1 and found that Staurosporine, but not Retro-2.1, blocked MERS-CoV (Fig 3A). Since Staurosporine and Retro-2.1 are active against SARS-CoV-2 in cells lacking TMPRSS2 our data suggest that they are not targeting TMPRSS2. In addition, Staurosporine is active against MERS-CoV, which may suggest a more general step in entry used by both coronaviruses or that Staurosporine is targeting multiple host factors that are independently utilized by both viruses.

### Staurosporine and Retro-2.1 do not impact ACE2 levels

Given that Staurosporine and Retro-2.1 block SARS-CoV-2 entry in TMPRSS2-expressing and TMPRSS2-deficient cells, we sought to determine the effects of these compounds on ACE2 and TMPRSS2 mRNA levels. Calu-3 cells were treated with DMSO vehicle control and each compound for 24 hours and subject to RT-qPCR. ACE2 mRNA was not affected by TMPRSS2 inhibitors, endosomal inhibitors, Staurosporine, or Retro-2.1 treatments compared to DMSO control treatment (S5A Fig). We found that Staurosporine and Retro-2.1 treatment reduced TMPRSS2 mRNA expression ~2-fold (S5B Fig**)**. Since these drugs are active in A549-ACE2 and Huh7.5 cell models that are not thought to express TMPRSS2, we examined the levels of TMPRSS2 in these cells. Indeed, we confirmed that these cells do not express TMPRSS2 (S5C-S5D Fig). Next, we monitored ACE2 expression by immunoblot. Treatment with TMPRSS2 enzymatic inhibitors, but not endosomal inhibitors Aloxistatin and Apilimod, appeared to increase ACE2 protein levels compared to vehicle control, suggesting ACE2 is a substrate of TMPRSS2 (S5E Fig) [52,53]. However, quantification of band intensities demonstrate no significant changes in ACE2 expression (S5F Fig). Similarly, quantification of Staurosporine and Retro-2.1 treatments resulted in no significant changes in ACE2 expression (S5F Fig). Since both Staurosporine and Retro-2.1 have antiviral activity in cell types that rely on endosomal entry, we treated A549-ACE2 cells with each compound and performed immunoblot analysis. Treatment with TMPRSS2 enzymatic inhibitors, Staurosporine, and Retro-2.1 had no effect on ACE2 protein expression (S5G-S5H Fig). Overall, our data suggest the antiviral activity of Staurosporine and Retro-2.1 is not due to negative regulation of ACE2 or TMPRSS2 levels.

### Staurosporine and Retro-2.1 inhibit entry at distinct steps

Given that Staurosporine and Retro-2.1 block SARS-CoV-2 entry, we sought to determine the step in the entry pathway at which inhibition occurs. We utilized a biochemical approach to determine if the inhibitors impact SARS-CoV-2 binding to Calu-3 cells. Calu-3 cells were treated with DMSO vehicle control, Staurosporine, and Retro-2.1 for one hour prior to SARS-CoV-2 binding (MOI 2) at 4°C. After one hour, cells were washed with PBS to remove unbound virus and total RNA was purified to detect bound virions. As a positive control, we used trypsin to remove bound virus and we confirmed significant reduction in binding compared to non-trypsinized, vehicle treated cells (Fig 3B). Furthermore, we found that treatment with Camostat, Staurosporine, or Retro-2.1 had no effect on SARS-CoV-2 binding compared to DMSO control (Fig 3B).

Next, we developed an entry bypass assay to determine if our inhibitors function upstream or downstream of Spike cleavage. We took advantage of the fact that Camostat blocks TMPRSS2 from cleaving SARS-CoV-2 Spike during entry, but that this can be bypassed by treating cells exogenously with the protease trypsin, which can cleave and activate Spike fusion [54]. Therefore, compounds that are bypassed by trypsin, such as Camostat, function either at or before the Spike cleavage step of SARS-CoV-2 entry. In contrast, compounds that are not affected by trypsin function downstream of this step. Calu-3 cells were treated with DMSO, Camostat, Staurosporine, or Retro-2.1 in duplicate one hour prior to binding (MOI 2) at 4°C. After one hour, cells were washed with PBS to remove unbound virus and either incubated with TPCK-treated trypsin to promote Spike cleavage and viral entry, or incubated with media, at 37°C for 5 minutes. Cells were then washed and incubated with the respective compounds for 20 hours prior to collection. As expected, Camostat, Staurosporine, and Retro-2.1 blocked SARS-CoV-2 viral RNA replication relative to DMSO **(**Fig 3C). Treatment with TPCK-treated trypsin resulted in increased infection of control cells, demonstrating that the addition of trypsin enhances Spike cleavage and infection (Fig 3C). As expected, Camostat is no longer antiviral when TPCK-treated trypsin is added (**Fig 3C**). Similarly, the antiviral activity of Staurosporine was abrogated by the addition of trypsin **(**Fig 3C**)**. In contrast, Retro-2.1 remains antiviral in the presence of exogenous trypsin (**Fig 3C**). Altogether, both Camostat and Staurosporine are bypassed by trypsin, suggesting that they function at or upstream of Spike cleavage during SARS-CoV-2 entry. Alternatively, the antiviral activity of Retro-2.1 is not bypassed by trypsin, and therefore, likely functions at a step downstream of Spike cleavage, supporting a role downstream of ACE2 engagement.

### PKCη is proviral during SARS-CoV-2 entry

We next sought to determine the host target of Staurosporine required for SARS-CoV-2 infection. Staurosporine is a well-established inhibitor of all PKC isozymes [46]. Interestingly, Ingenol Mebutate and PMA also impact PKC, and were among the 13 entry inhibitors identified by the VSV screen (Fig 1A-1B**)**. Thus, we hypothesized that PKC promotes SARS-CoV-2 entry. PKCs have been shown to be activated and/or play a role during entry of several viruses including Rift Valley fever virus, adenovirus, influenza, rhabdovirus, alphavirus, poxvirus, herpesvirus, and respiratory syncytial virus [55–59]. Furthermore, the PKCβ isozyme has been implicated to function during SARS-CoV-2 entry [60]. Although we found Staurosporine to be an inhibitor of SARS-CoV-2 entry, we also observed modest cytotoxic effects (Fig 1A-1B). Thus, we tested five additional PKC inhibitors with different selectivity’s (Rottlerin, Enzastaurin, Go 8963, Go 6976, and Chelerythrine Chloride) and found that none of these PKC inhibitors impacted SARS-CoV-2 infection of Calu-3 cells (S6A-S6B Fig).

**Fig 4:**
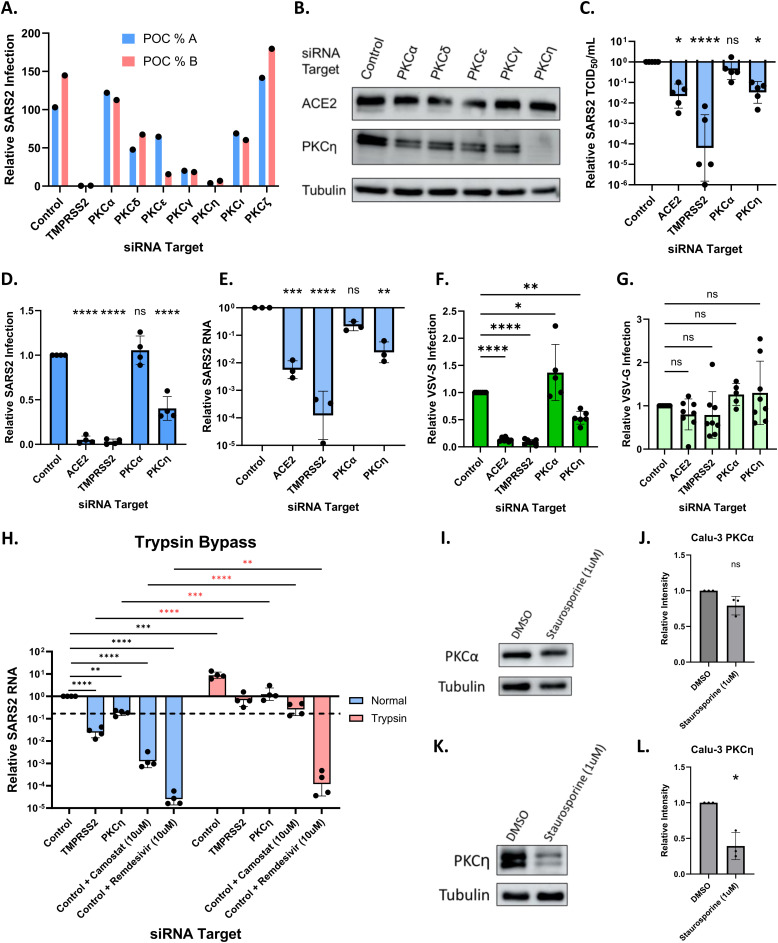
PKCη is Proviral During SARS-CoV-2 Entry. **(A)** Calu-3 cells were transfected with the indicated siRNA and infected at 48 hours with SARS-CoV-2 (MOI 0.5) for 48 hours followed by automated microscopy and image analysis quantifying total cell numbers (nuclei+) and percent infection (Spike + /nuclei+). Screen was performed in duplicate (blue and red). **(B)** Calu-3 cells were transfected with the indicated siRNA for 48 hours and cell lysates were collected for immunoblot with the indicated antibodies. Representative blots are shown for n = 3. **(C)** Calu-3 cells were transfected with the indicated siRNA and infected at 48 hours with SARS-CoV-2 (MOI 0.2) for 48 hours prior to supernatant collection. VeroTMPRSS2 cells were infected with serial dilutions of supernatants for 48 hours to determine viral titers by TCID_50_. **(D)** Calu-3 cells were transfected with the indicated siRNA and infected at 48 hours with SARS-CoV-2 (MOI 0.2) for 48 hours prior to processing for automated microscopy and image analysis quantifying total cell numbers (nuclei+) and percent infection (Spike + /nuclei+). **(E)** Calu-3 cells were transfected with the indicated siRNA and infected at 48 hours with SARS-CoV-2 (MOI 0.2) for 48 hours prior to processing for RT-qPCR analysis of viral RNA (Nucleocapsid). **(F)** Calu-3 cells were transfected with the indicated siRNA and infected at 48 hours with VSV-S (MOI 6) for 24 hours prior to processing for automated microscopy and image analysis quantifying total cell numbers (nuclei+) and percent infection (GFP + /nuclei+). **(G)** Calu-3 cells were transfected with the indicated siRNA and infected at 48 hours with VSV-G (MOI 0.04) for 24 hours prior to processing for automated microscopy and image analysis quantifying total cell numbers (nuclei+) and percent infection (GFP + /nuclei+). **(H)** Calu-3 cells were transfected with the indicated siRNA for 48 hours, pre-treated with compounds where indicated, and infected with SARS-CoV-2 (MOI 1 or 0.2) for 30 minutes at 4°C. Cells were washed with PBS to remove unbound virus and either fresh media or 10µg/mL of TPCK-treated trypsin was added and incubated for five minutes at 37°C. Cells were then washed and replaced with fresh media and compounds for 20 hours. Cell lysates were processed for RT-qPCR analysis of viral RNA (Nucleocapsid). A dashed line is drawn at the mean of PKCη to visualize the bypass by TPCK-treated trypsin. **(I)** Calu-3 cells were treated with vehicle control (DMSO) or Staurosporine 1uM for 24 hours and cell lysates were collected for immunoblot with PKCα antibody. Representative blots are shown for n = 3. **(J)** Band intensity quantification of PKCα levels normalized to Tubulin analyzed by unpaired t test with Welch Correction. **(K)** Calu-3 cells were treated with vehicle control (DMSO) or Staurosporine 1uM for 24 hours and cell lysates were collected for immunoblot with PKCη antibody. Representative blots are shown for n = 3. **(L)** Band intensity quantification of PKCη levels normalized to Tubulin analyzed by unpaired t test with Welch Correction. Means + SD for individual biological replicates are shown. Significance for relative TCID_50_/mL, infection, and viral RNA was calculated using One Way ANOVA with Dunnett Correction on controls and significance for relative viral RNA of the trypsin bypass was calculated using One Way ANOVA with Sidak Correction for multiple comparisons (*p < 0.05, **p < 0.01, ***p < 0.001, ****p < 0.0001; ns, no significance.

While Staurosporine can block all PKCs, these drugs cannot block every isozyme. Therefore, we set out to determine which PKC isozyme may be required for SARS-CoV-2 entry. We first mined our RNAseq data for transcript levels of each PKC isozyme in Calu-3 cells and found that seven were expressed, while PKCβ was not (S7A Fig**)** [22]. We then utilized siRNAs to deplete each expressed isozymes to determine the impact on SARS-CoV-2 infection in duplicate. As a positive control, we depleted TMPRSS2 and observed strong reduction in infection along with depletion of the conventional and novel PKCγ and PKCη isozymes, respectively (Fig 4A). To determine if PKCγ or PKCη promote infection outside of the screen, we tested pooled and individual siRNAs during infection. None of the siRNAs targeting PKCγ efficiently deplete PKCγ by RT-qPCR and only one blocked infection demonstrating off-target effects (S7B-S7C Fig). Alternatively, multiple siRNAs targeting PKCη blocked infection, and efficiently depleted PKCη **(**S7B-S7D Fig**)**. Immunoblot analysis confirmed robust expression and depletion of PKCη **(**Figs 4B and S7E). We also monitored ACE2 levels upon depletion of PKC isozymes and found no effect, suggesting that PKC does not control ACE2 levels and that PKCη blocks entry by a distinct mechanism **(**Fig 4B**)**. To further characterize the proviral phenotype, we depleted ACE2, TMPRSS2, PKCα, and PKCη or control and infected these cells with SARS-CoV-2 for 48 hours and assayed infection by viral titer, microscopy and RT-qPCR. As expected, depletion of ACE2 and TMPRSS2 blocked infection (Fig 4C-4E). Depletion of the conventional PKCα had no impact on infection whereas depletion of PKCη attenuated infection **(**Fig 4C-4E**)**. Furthermore, depletion of each of these genes had no impact on cell viability (S7F Fig). We further confirmed that PKCη promoted infection of SARS-CoV-2 Omicron BA.1 and BA.5 variants by both microscopy and RT-qPCR assays (S7G-S7J Fig).

Next, we tested whether PKCη promotes entry by comparing its effect on VSV-S to VSV-G infection. We depleted ACE2, TMPRSS2, PKCα, and PKCη and infected with either VSV-S or VSV-G and quantified infection. ACE2 and TMPRSS2 depletion inhibited VSV-S but not VSV-G (Fig 4F-4G). Depletion of PKCη also reduced infection of VSV-S while having no impact on VSV-G (Fig 4F-4G). This suggests that PKCη is involved in SARS-CoV-2 entry.

Staurosporine blocks entry upstream of TMPRSS2 (Fig 3C) and so we hypothesized that PKCη would also be required at this step. Therefore, we performed our trypsin bypass assay where we depleted TMPRSS2 or PKCη and infected these or control depleted cells with SARS-CoV-2. In addition, Camostat was used as a treatment that can be bypassed, while Remdesivir, an RdRp inhibitor blocking RNA replication, was used as a treatment that cannot be bypassed. Under normal conditions, depletion of TMPRSS2 and PKCη or treatment with Camostat and Remdesivir resulted in significant reduction infection (Fig 4H). Treatment with TPCK-treated trypsin increased infection of control cells ~10-fold and resulted in bypass of TMPRSS2, PKCη, and Camostat, but not Remdesivir, which remains highly active (Fig 4H). This suggests that PKCη functions at or upstream of Spike cleavage.

We further explored the connection between PKCη and Staurosporine. First, we determined if Staurosporine treatment impacted the levels of PKCη. Indeed, Staurosporine treatment led to a significant reduction of PKCη, but not PKCα as measured by immunoblot (Fig 4I-4LFigure 4JFigure 4KFigure 4L). Moreover, if Staurosporine targets PKCη the loss of PKCη would shift the IC_50_ of Staurosporine. To test this we transfected Calu-3 cells with non-targeting siRNA or siRNA targeting PKCη and determined the IC_50_ for Staurosporine under both conditions. Depletion of PKCη reduced the IC_50_ for Staurosporine by 0.017-fold compared to non-targeting siRNA control (S7K Fig). Overall, these data suggest that Staurosporine inhibits SARS-CoV-2 entry through inhibition of PKCη. Given that Staurosporine blocked MERS-CoV infection, we sought to determine if PKC depletions would also block infection. We depleted TMPRSS2 or the PKC isozymes for 48 hours and infected cells with MERS-CoV (MOI 0.2) for 24 hours. As expected, TMPRSS2 depletion blocked infection **(**S7L Fig**)**. However, depletion of the PKCs had no effect on infection **(**S7L Fig**)**. This suggests that PKCη selectively functions at or before the Spike cleavage step to promote SARS-CoV-2 entry and blocks MERS by a distinct mechanism.

### Retro-2.1 SAR studies suggest NF1 targeting impacts SARS-CoV-2

**Fig 5:**
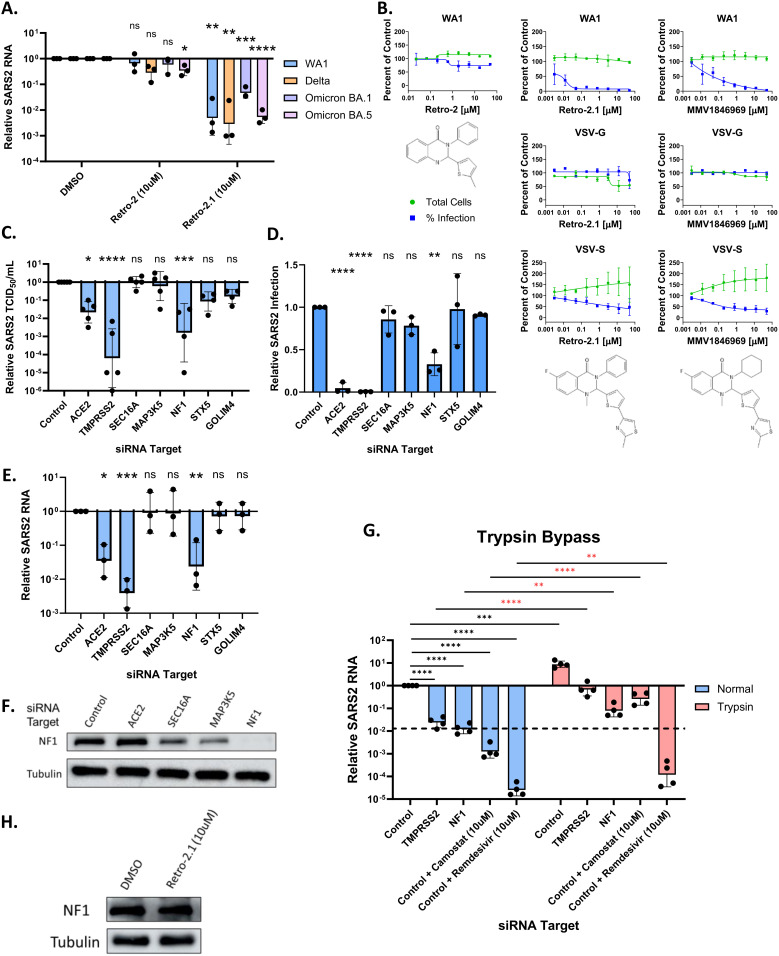
NF1 promotes SARS-CoV-2 infection. **(A)** Calu-3 cells were pre-treated with vehicle control (DMSO), Retro-2 (10µM), or Retro-2.1 (10µM) and infected with SARS-CoV-2 variants (MOI 0.2) for 48 hours prior to processing for RT-qPCR analysis of viral RNA (Nucleocapsid). **(B)** Calu-3 cells were pre-treated with the indicated compounds in 8-point dose-response and infected with SARS-CoV-2 (MOI 0.5; 48 hours), VSV-G (MOI 0.05; 24 hours), or VSV-S (MOI 5; 24 hours) followed by automated microscopy and image analysis quantifying total cell numbers (green) (nuclei+) and percent infection (blue) (Spike + , dsRNA + , or GFP + /nuclei+). Data are presented as mean values of n = 2 independent biological replicates ± SD. **(C)** Calu-3 cells were transfected with the indicated siRNA and infected at 48 hours with SARS-CoV-2 (MOI 0.2) for 48 hours prior to supernatant collection. VeroTMPRSS2 cells were infected with serial dilutions of supernatants for 48 hours to determine viral titers by TCID_50_. **(D)** Calu-3 cells were transfected with the indicated siRNA and infected at 48 hours with SARS-CoV-2 (MOI 0.2) for 48 hours prior to processing for automated microscopy and image analysis quantifying total cell numbers (nuclei+) and percent infection (Spike + /nuclei+). **(E)** Calu-3 cells were transfected with the indicated siRNA and infected at 48 hours with SARS-CoV-2 (MOI 0.2) for 48 hours prior to processing for RT-qPCR analysis of viral RNA (Nucleocapsid). **(F)** Calu-3 cells were transfected with the indicated siRNA for 48 hours and cell lysates were collected for immunoblot with the indicated antibodies. Representative blots are shown for n = 3. **(G)** Calu-3 cells were transfected with the indicated siRNA for 48 hours, pre-treated with compounds where indicated, and infected with SARS-CoV-2 (MOI 1 or 0.2) for 30 minutes at 4°C. Cells were washed with PBS to remove unbound virus and either fresh media or 10µg/mL of TPCK-treated trypsin was added and incubated for five minutes at 37°C. Cells were then washed and replaced with fresh media and compounds for 20 hours. Cell lysates were processed for RT-qPCR analysis of viral RNA (Nucleocapsid). A dashed line is drawn at the mean of NF1 to visualize the inhibitory effect under TPCK-treated trypsin conditions. Means + SD for individual biological replicates. **(H)** Calu-3 cells were treated with the indicated compounds for 24 hours and cell lysates were collected for immunoblot with the indicated antibodies. Significance for relative viral RNA was calculated using One Way ANOVA with Dunnett Correction on DMSO control and significance for relative viral RNA of the trypsin bypass was calculated using One Way ANOVA with Sidak Correction for multiple comparisons (*p < 0.05, **p < 0.01, ***p < 0.001, ****p < 0.0001; ns, no significance.

Retro-2.1 was identified as a more potent small molecule derivative of Retro-2, which inhibits retrograde trafficking [47,48]. Indeed, Retro-2 and Retro-2.1 both inhibit retrograde trafficking of bacterial toxins through binding to the ER exit site protein, SEC16A, and reducing anterograde transport of the SNARE protein STX5 [47,48]. This prevents STX5 interaction with GOLIM4, which plays a role in endosome to Golgi trafficking, altogether blocking retrograde transport of bacterial toxins [48]. Retro-2 and Retro-2.1 inhibit trafficking steps of several pathogens including blocking entry, replication, or packaging/egress of several viruses [61–67]. Retro-2.1 was also previously implicated in blocking SARS-CoV-2 infection at a post-entry step [68]. When we tested Retro-2, we found that it is inactive against SARS-CoV-2, suggesting that Retro-2.1 is inhibiting a distinct host target from the previous studies (Fig 5A-5B). To further explore this, we conducted structure-activity relationship (SAR) studies by testing 17 additional derivatives of Retro-2.1 against SARS-CoV-2, VSV-G, and VSV-S. We identified 9 derivatives with an SI > 10 against SARS-CoV-2, of which only one (MMV1846969) had an SI > 10 against VSV-S **(**Figs 5B and S8, S3 Table**)**. Of the 17 derivatives tested, one (MMV1846973) has previously been reported to be more potent than Retro-2 against Shiga toxin, and we found that it is active against SARS-CoV-2, but not on VSV-S suggesting that there are distinct activities [69].

Given that we found that Retro-2.1, but not Retro-2, displayed antiviral activity against SARS-CoV-2 and VSV-S, we hypothesized that Retro-2.1 acts through an alternative target than SEC16A during SARS-CoV-2 entry. SEC16A was originally identified by proteomics of Retro-2.1 cellular binders. Within that screen, there were two additional candidates, MAP3K5 and NF1 [48]. Therefore, we set out to determine if SARS-CoV-2 infection was sensitive to SEC16A and its binding partners STX5 or GOLIM4, and found that these depletions were not cytotoxic (S9A Fig). Next, we depleted each gene, infected with SARS-CoV-2, and quantified infection by viral titer, microscopy or RT-qPCR. While depletion of ACE2 and TMPRSS2 reduced infection, depletion of SEC16A, STX5, or GOLIM4 did not **(**Fig 5C-5E**)** [48]. Next, we tested the other Retro-2.1 binders NF1 and MAP3K5 and found depletions were not cytotoxic, and that loss of NF1 but not MAP3K5 led to decreased infection by viral titer, microscopy, and RT-qPCR **(**S9A Fig **and 5C-5E)**. We also confirmed the pro-viral phenotype of NF1 against the Omicron BA.1 and Omicron BA.5 variants in Calu-3 cells **(**S9B-S9E Fig**)**. Next, we confirmed expression and efficient depletion of NF1 in Calu-3 cells with immunoblotting following 48-hour transfections with non-targeting siRNA control and siRNAs targeting ACE2, SEC16A, MAP3K5, and NF1 **(**Figs 5F and S9G**)**. Although depletion of SEC16A had no effect on SARS-CoV-2 infection, it modestly reduced NF1 expression (Figs 5F and S9F**)**.

To determine if NF1 functions downstream of TMPRSS2 cleavage during entry, similar to Retro-2.1, we conducted a trypsin bypass experiment during depletion of TMPRSS2 and NF1. We also included Camostat as a positive control for bypass and Remdesivir as an RdRp inhibitor that cannot be bypassed. Depletion of TMPRSS2 and NF1 resulted in significant reduction of infection in addition to Camostat and Remdesivir treatments **(**Fig 5G**)**. Addition of TPCK-treated trypsin showed an increase in infection of control cells and resulted in significant bypass of Camostat with ~100-fold increase in infection **(**Fig 5G**)**. The addition of TPCK-treated trypsin resulted in a minor increase in infection during NF1 depletion and Remdesivir treatment, however, both conditions continued to inhibit infection **(**Fig 5G**)**. Altogether, these data suggest that NF1 functions downstream of the Spike cleavage step to promote SARS-CoV-2 infection. We also tested if Retro-2.1 impacted levels of NF1 and as expected observed no differences in expression compared to DMSO control **(**Fig 5H**)** [48]. NF1 is known to negatively regulate the Ras signaling pathway, therefore, we explored the effects of Ras activation on infection [70,71]. We pre-treated Calu-3 cells with PBS control or EGF to induce the Ras pathway and infected with SARS-CoV-2 for 48 hours. We found that EGF treatment significantly reduced infection, suggesting that Ras signaling inhibits SARS-CoV-2 infection **(**S9H**)**. Since Retro-2.1 was also active in A549-ACE2 cells where SARS-CoV-2 utilizes endosomal entry, we investigated which of the three cellular binders promotes infection. We depleted SEC16A, MAP3K5, NF1, ACE2, and RAB7A as an additional positive control for inhibition of endosomal trafficking, and infected with SARS-CoV-2 for 24 hours. As expected, depletion of ACE2 and RAB7A blocked infection in A549-ACE2 cells **(**S9I Fig**)**. Interestingly, we found that depletion of SEC16A, but not NF1, blocked infection in A549-ACE2 cells **(**S9I Fig**)**. Thus, NF1 promotes infection in Calu-3 cells during TMPRSS2-dependent entry whereas SEC16A promotes infection in A549-ACE2 cells during TMPRSS2-independent entry.

## Discussion

We used a two-step screening strategy to identify inhibitors of SARS-CoV-2 entry. We found 13 candidates that selectively blocked SARS-CoV-2 and VSV-S infection without impacting VSV-G. These included the canonical TMPRSS2 inhibitors, Camostat and Nafamostat, in addition to Avoralstat and UK-371804, serine protease inhibitors recently described as inhibitors of TMPRSS2 and SARS-CoV-2 entry [43,44]. We also identified the pan-PKC inhibitor Staurosporine along with two other compounds that regulate PKC, Ingenol Mebutate and PMA, as inhibitors of SARS-CoV-2 entry. Additionally, we identified Retro-2.1 as a potent entry inhibitor. Thus, we set out to determine the mechanism of action of these inhibitors and their host targets.

We found that Staurosporine blocked entry at or prior to the Spike cleavage step but did not affect viral binding. Furthermore, Staurosporine was active in cells that use endosomal entry suggesting a role independent of TMPRSS2. PKCs are a large family of kinases and more specific PKC inhibitors have been developed that can block the activity of particular PKCs. However, none of the characterized specific PKC inhibitors blocked SARS-CoV-2 infection, suggesting the involvement of a specific PKC isozyme in SARS-CoV-2 entry [46]. PKCs regulate receptor internalization, membrane trafficking, and endocytosis [30–34,72]. Several studies have established a role for PKCs during entry of various viruses, including internalization, receptor-mediated endocytosis, endosomal trafficking, and membrane fusion [55–59]. Moreover, PKC inhibition has been shown to block SARS-CoV-2 entry, with PKCβ suggested as the proviral target [60]. However, the panel of PKC isozymes tested against SARS-CoV-2 was limited and PKCβ was not expressed in our cells while seven other PKC isozymes were. Using genetic screening and validation we found that PKCη promotes infection. PKCη is a novel isozyme predominantly expressed in epithelial tissues including the respiratory tract [73]. Similar to Staurosporine, we found that PKCη was proviral during SARS-CoV-2 entry at or prior to Spike cleavage. Further, PKCη depletion had no effect on MERS-CoV, suggesting a specific role for SARS-CoV-2 entry.

Our data support a role for PKCη involvement during SARS-CoV-2 entry at or prior to Spike cleavage without affecting ACE2 expression. Thus, we propose PKCη influences SARS-CoV-2 entry after viral binding but prior to internalization. One possibility is that PKCη regulates localization of virions into cell membrane microdomains by tetraspanins, as it has been shown that inhibition of these microdomains attenuates viral entry [74–77]. Alternatively, studies have shown that PKCη can regulate tight junctions through phosphorylation of occludin [78]. Occludin can promote SARS-CoV-2 internalization and cell-to-cell transmission during endosomal (TMPRSS2-independent) entry [79–81]. Moreover, depletion of occludin was also shown to reduce SARS-CoV-2 internalization, but not binding, in Caco-2 cells expressing endogenous ACE2 and TMPRSS2 (TMPRSS2-dependent entry) [79]. Therefore, PKCη may regulate occludin-mediated entry, enabling viral internalization. Future studies will determine the precise mechanism by which PKCη promotes SARS-CoV-2 entry.

We also identified the retrograde trafficking inhibitor Retro-2.1 as an inhibitor of SARS-CoV-2 entry. Although Retro-2 and Retro-2.1 have been shown to block entry, replication, and egress of several viruses, we found Retro-2 to be inactive against SARS-CoV-2 [61–67]. We found that Retro-2.1 does not affect viral binding and blocks infection at a previously unrecognized step downstream of Spike cleavage in TMPRSS2 expressing cells. Since Retro-2.1 also blocks infection in cells lacking TMPRSS2, our data suggest that there is a common downstream step in the entry pathway between surface TMPRSS2-dependent entry and endosomal entry. This led us to explore the host target of Retro-2.1. The best characterized target of Retro-2.1 (and Retro-2) is SEC16A, which we found had no impact on infection in Calu-3 cells. Therefore, we mined the chemoproteomic study that identified Retro-2.1 binders for additional targets and found that depletion of NF1 reduced infection. Similar to Retro-2.1, NF1 functions downstream of Spike cleavage during SARS-CoV-2 infection. Alternatively, depletion of SEC16A in A549-ACE2 cells reduced infection, demonstrating that Retro-2.1 likely binds multiple targets that are cell-type dependent for SARS-CoV-2 infection. Altogether, our data suggest NF1 is an alternative target of Retro-2.1 that is involved following Spike cleavage during TMPRSS2-dependent SARS-CoV-2 infection of Calu-3 cells.

NF1 encodes the ubiquitously expressed neurofibromin, a tumor suppressor involved in regulating diverse cellular processes [71,82]. NF1 contains multiple domains and the GAP-related domain (GRD), is the most studied and an established negative regulator of the Ras signaling pathway [70,71]. Interestingly, NF1 promotes alphavirus infection through Ras signaling [83]. Although the connection between NF1 and SARS-CoV-2 infection remains unclear, Ras signaling may affect pathways involved in SARS-CoV-2 infection. Interestingly, NF1 also contains phosphorylation sites that can be acted upon by PKA and PKC [84,85]. PKC phosphorylation has been shown to contribute to the negative regulation of Ras signaling and increase association with actin [85]. NF1 is also known to regulate cytoskeletal organizations by interacting with microtubules, actin filaments, and intermediate filaments [86,87]. Importantly, NF1 has been shown to directly interact or be in complex with proteins involved in anterograde and retrograde transport along microtubules, suggesting a role for intracellular trafficking [71,88–90]. Our data suggest Retro-2.1 targets NF1 downstream of Spike cleavage at a late stage of viral entry or an early post-entry step of SARS-CoV-2 infection, potentially through Ras-mediated cytoskeletal alterations or inhibition of intracellular trafficking. Future studies will determine how NF1 promotes SARS-CoV-2 entry.

While it is clear that SARS-CoV-2 entry initiates with Spike binding to ACE2, there are likely additional steps involved. The apical expression of TMPRSS2 in host cells dictates which entry pathway is utilized, ultimately resulting in the release of the viral genome into the cytoplasm for viral replication. However, the full spectrum of proteins involved in SARS-CoV-2 entry is incompletely understood. The identification of additional host factors such as PKCη and NF1 that impact both entry pathways may elucidate requirements of membrane or cytoskeletal organization required for viral internalization or intracellular trafficking.

## Materials and methods

### Cells and viruses

Human adenocarcinoma lung epithelial Calu-3 cells (American Type Culture Collection HTB-55) were cultured in minimum essential medium (MEM) supplemented with 10% (v/v) fetal bovine serum, 1% (v/v) nonessential amino acids, 1% (v/v) penicillin/streptomycin, and 1% (v/v) Glutamax (Invitrogen). Caco-2 cells (ATCC, HTB-37) were cultured in MEM alpha supplemented with 20% (v/v) fetal bovine serum, 1% (v/v) penicillin–streptomycin and 1% (v/v) l-glutamine. Plates were coated with rat tail collagen (Corning) for 30 min and washed in PBS before plating and used for all Calu-3 and Caco-2 experiments. A549-ACE2 cells were cultured in RPMI-1640, 10% fetal bovine serum (FBS), 1% penicillin/streptomycin, and 1% Glutamax. Huh7.5 cells (C. Rice, Rockefeller) were cultured in DMEM supplemented with 10% (v/v) fetal bovine serum, 1% (v/v) penicillin/streptomycin and 1% (v/v) Glutamax. iAT2 cells were differentiated from the SPC2 iPSC line, clone SPC2-ST-B2 (Boston University) and maintained as alveolospheres. The alveolospheres were dissociated into single cells and plated on 3% Matrigel coated plates in CK + DCI media supplemented with 2 μM TZV for two days. Culture medium was changed to CK + DCI media 3 days post-plating prior to SARS-CoV-2 infection [38]. All cells were grown at 37 °C, 5% CO_2_ and 20% O_2_. Cells were validated to be mycoplasma free using MycoStrip Mycoplasma Detection Kit (InvivoGen).

SARS-CoV-2 was obtained from Andrew Pekosz and BEI Resources; USA WA1/2020 strain (Cat# NR-52281), Alpha Isolate hCoV-19/England/204820464/2020 lineage B.1.1.7 (Cat# NR-54971), Beta Isolate hCoV-19/USA/MD-HP01542/2021 Lineage B.1.351 (Cat# NR-55282), Delta Isolate hCoV-19/USA/MD-HP05285/2021, Omicron BA.1 Isolate hCoV-19/USA/MD-HP20874/2021 Lineage B.1.1.529 (Cat# NR-56461), and Omicron BA.5 Isolate hCoV-19/USA/COR-22–063113/2022 Lineage BA.5 (Cat# NR-58616). Viral stocks were prepared by infection of Vero-TMPRSS2 cells in 2% serum and 10 mM Hepes for three days, freeze-thawed, and clarified by centrifugation at 3,000 rpm for 20 minutes. Supernatant containing virus was aliquoted and stored at −80°C (P0). The seed stock (P0) was sequence verified, amplified in Vero-TMPRSS2 cells (P1), and used for all experiments. Virus stock titers were determined by 50% tissue culture infective doses (TCID_50_) using the Reed-Muench method in Vero-TMPRSS2 cells [16]. rMERS-CoV was a gift from Ralph Baric. All work with SARS-CoV-2 and MERS-CoV was performed in a biosafety level 3 (BSL-3) laboratory and approved by the Institutional Biosafety Committee and Environmental Health and Safety. Vesicular stomatitis virus (VSV-G and VSV-S) were a gift from Sean Whelan.

### Screens and automated microscopy

Automated microscopy experiments were performed as described previously [16]. Pandemic Response Box Screening: Calu-3 cells (8.0 x 10^3^/well) were plated in 384 well plates (Corning Biocoat) in growth medium. The following day, 50nL of compounds were added to assay plates to achieve a final concentration of 10uM in 0.2% DMSO. 10uM remdesivir (n = 32) and 0.2% DMSO (n = 32) was added to each assay plate as a positive and negative control, respectively. After one hour, cells were infected with SARS-CoV-2 (MOI 0.5) for 48 hours. Cells were fixed with 4% formaldehyde for 15 minutes at room temperature and washed three times with PBS. Cells were then blocked in 2% BSA PBST for one hour and incubated in primary antibody (anti-dsRNA J2) overnight at 4°C. Following three PBST washes, cells were incubated in secondary antibody (anti-mouse Alexa 488) and Hoescht 33342 for one hour at room temperature. Cells were washed 3x in PBST and imaged at 10X using ImagXpress Micro capturing four sites per well. The total number of cells (nuclei+) and the number of dsRNA+ cells were measured using cell scoring module (MetaXpress 6.7.0), and the percentage of infected cells (dsRNA + /nuclei+) was calculated. Sample well infection was normalized to aggregated DMSO plate control wells and expressed as percentage of control (POC = (% infection_sample_/average % infection_DMSO_) × 100) and *Z* score (*Z* = (% Infection_sample_ − average % Infection_DMSO_)/Standard Deviation %infection_DMSO_) in Spotfire (revvity).

VSV Entry Assay: Calu3 cells (8.0 x 10^3^/well) were plated in 384 well plates (Corning Biocoat) in growth medium. Compounds arrayed as an 8-pt dose-response were added to assay plates to achieve final concentrations in 0.2% DMSO. Cells were infected 1 hour post-treatment with VSV-S MOI = 5 or VSV-G MOI = 0.05 for 24 hours to achieve similar levels of infection (20–50% of control). Cells were fixed and processed for microscopy as described and percent infection (GFP + /nuclei) and cell number (nuclei+) was quantified. A non-linear regression curve fit analysis (GraphPad Prism 9) was performed on the aggregated average POC Infection and cell viability from ≥ 2 independent experimental replicates versus the log_10_ transformed concentration values to calculate IC_50_ values for Infection and CC_50_ values for cell viability for each drug/cell line combination. Error bars represent the standard deviation of replicate data for each drug concentration tested in independent experiments. Selectivity index (SI) was calculated as a ratio of drug’s CC_50_ and IC_50_ values (SI = CC_50_/IC_50_).

Validation studies: Calu-3 cells (4.5 X 10^4^/well) were plated on collagen coated 96 well plates (Corning BioCoat) and treated as indicated in triplicate. Cells were fixed with 4% formaldehyde for 15 minutes at room temperature and washed three times with PBS. Cells were permeabilized and blocked using 2% BSA in PBS-T for one hour at room temperature. Plates were incubated overnight with primary antibody (anti-SARS-CoV-2 spike) (Sotrovimab), (anti-SARS-CoV-2 nucleocapsid) (GeneTex), or (anti-dsRNA J2) (Millipore) at 4°C, washed three times with PBS-T, and incubated with Alexa Flour conjugated secondary antibodies (anti-Human Alexa 594, anti-Rabbit Alexa 594, or anti-Mouse Alexa 488) and Hoechst 33342 for one hour at room temperature. After three washes, plates were sealed and imaged using an automated microscope (ImageXpress Micro, Molecular Devices). Cells were imaged with a 10 × objective, and nine sites were captured per well. The total cell numbers and the infected cell numbers were measured using the cell scoring software (MetaXpress 6.7.0), and the percentage of infected cells was calculated.

Cell viability studies: Cells were treated with compounds continuously for 72 hours and metabolic viability was determined by ATPlite (revvity). Sample well data was normalized to aggregate DMSO control well data and expressed as percentage of control (POC). A non-linear regression curve fit analysis of POC viability versus the log10 transformed drug concentration was used to determine the IC50 values. Antibodies used in this study are listed in **S4 Table**.

### siRNA transfections

Genes of interest were depleted by reverse transfection using siRNAs with Lipofectamine RNAiMax reagent (Thermo Scientific) according to the manufacturer’s protocol. Briefly, Calu-3 cells were plated on collagen coated 6 well plates (5 X 10^5^) or collagen coated 96 well plates (4.5 X 10^4^) (Corning BioCoat) and transfected with 25nM siRNAs with Lipofectamine RNAiMax for 48 hours. Cells were infected or treated as indicated and collected at the indicated time point with either TriZol to analyze RNA levels, RIPA buffer to be used for western blotting, or fixed with 4% formaldehyde for automated microscopy. Cell death siRNA (Qiagen) was used as a positive control for transfection efficiency. All siRNA sequences are listed in **S5 Table**.

### RNA isolation and RT-qPCR

Total RNA was purified using Trizol (Invitrogen) followed by RNA Clean and Concentrator-25 kit (Zymo Research). cDNA was synthesized from 1 µg of RNA using random hexamers, dNTP, and Moloney murine leukemia virus (M-MLV) reverse transcriptase (Invitrogen). Target genes were amplified using gene specific primers and SYBR green master mix (Applied Biosystems) and 18S rRNA primers were used to amplify endogenous control by using the QuantStudio 6 Flex RT-PCR system (Applied Biosystems). Relative quantities of viral and cellular RNA were calculated using the standard curve method [16,22]. Primers used for RT-qPCR are listed on **S6 Table**.

### Immunoblotting

Cells were washed with cold PBS and lysed in radioimmunoprecipitation assay (RIPA) buffer [50 mM tris-HCl, (pH 8.0), 150 mM NaCl, 0.5% sodium deoxycholate, 0.1% SDS, 1% NP-40, 1 mM PMSF, supplemented with protease and phosphatase inhibitor cocktails] [22]. Samples were centrifuged at 13,000 rpm for 15 minutes at 4°C and clarified cell lysates were quantified for protein levels using the BCA Protein Assay (ThermoScientific). Cell lysates were incubated in 5X sample buffer at 95°C for 10 minutes, run on 10% sodium dodecyl sulfate-polyacrylamide gel electrophoresis (SDS-PAGE) gels, and transferred to a PVDF membrane (Millipore). Membranes were blocked with 5% skim milk in tris-buffered saline with 0.1% Tween 20 (TBST) for one hour at room temperature and incubated with primary antibodies overnight at 4°C. Following three TBST rinses, the membranes were incubated with horseradish peroxidase (HRP)–conjugated secondary antibodies for one hour at room temperature. Membranes were washed three times with TBS-T and developed with West Femto Substrate (Thermofisher) and ECL Western blotting reagents (Amersham) before imaging with Amersham Imager 680 (Amersham). Antibodies used in this study are listed in **S4 Table**.

### SARS-CoV-2 binding and trypsin bypass

Calu-3 cells were plated on collagen coated 6 or 12 well plates and reverse transfected for 48 hours as described prior to infection as indicated. For drug treatments, Calu-3 cells were treated with vehicle or the indicated compounds in DMSO with a final concentration of 0.2% DMSO for one hour prior to infection. SARS-CoV-2 bound to cells for 30 minutes at 4°C followed by a cold PBS wash. For the binding assay, samples were collected in Trizol for further processing. As a positive control, 0.25% trypsin was added to untreated cells and incubated at 37°C for 5–10 minutes to remove bound virus. Cell culture media was then added to inhibit trypsin, and virus was removed by centrifuging cells and aspirating the supernatant. For the trypsin bypass, either 10µg/mL of TPCK-treated trypsin (ThermoFisher) or media was added following binding, and plates were incubated for five minutes at 37°C to enable synchronized entry. Cells were then washed and replaced with fresh media and compounds. Twenty hours later, samples were collected in Trizol.

### Statistical analysis

Statistical analyses were performed by using Prism (GraphPad Software, 10). For RT-qPCR and additional automated microscopy studies, statistical significances were investigated by unpaired t test with Welch’s correction or ordinary one-way ANOVA with Dunnett’s multiple comparisons test. Adjusted *p* values are described by asterisks in figures: (^*^) for *p* < 0.05, (^**^) for *p* < 0.01, (^***^) for *p* < 0.001, and (^****^) for *p* < 0.0001. All relative values with compounds are normalized to DMSO control and siRNA transfections are normalized to non-targeting siRNA Control. For reanalysis of RNAseq data, raw Calu-3 fastq files were trimmed, counted, and aligned as previously described [22]. Transcript counts were collapsed to the gene level in R using tximport v1.34.0 and raw gene-level count values were accessed using the *counts* matrix. Then, raw gene-level count values were plotted for the protein kinase c family using ggplot2 v3.5.2.

## Supporting information

S1 Fig High-throughput Screening of the Pandemic Response Box Identifies SARS-CoV-2 Antivirals. (A) Calu-3 cells were seeded in 384 well plates and pre-treated the following day with DMSO vehicle control, 10uM of Remdesivir positive control, or 10uM of the Pandemic Response Box (400 compounds) for one hour prior to SARS-CoV-2 (MOI 0.5) infection. After 48 hours, cells were fixed with 4% formaldehyde and processed for imaging to determine Percent of Control (POC) % Positive for SARS-CoV-2 infection. (B) Calu-3 cells were seeded in 384 well plates and pre-treated the following day with DMSO vehicle control, 10uM of Remdesivir positive control, or 10uM of the Pandemic Response Box (400 compounds) for one hour prior to SARS-CoV-2 (MOI 0.5) infection. After 48 hours, cells were fixed with 4% formaldehyde and processed for imaging to determine total cell counts as a readout for cell viability. Compounds were screened in duplicate (RepA & RepB). Yellow = negative control; Green = positive control; Blue = sample. Sixteen compounds were found to reduce infection below 40% and maintain cell viability above 80%.
(TIF)

S2 Fig Entry Inhibitors Block SARS-CoV-2 Infection. (A) Calu-3 cells were pre-treated with endosomal entry inhibitors in 8-point dose-response for one hour prior to SARS-CoV-2 (48 hours; MOI 0.5), VSV-G (24 hours; MOI 0.05), or VSV-S (24 hours; MOI 5) infection. Cells were processed for automated microscopy and image analysis quantifying total cell numbers (green) (nuclei+) and percent infection (blue) (Spike+ or dsRNA + /nuclei+) or (GFP + /nuclei+) to determine Percent of Control (POC) % Positive. Data are presented as mean values of n = 2 independent biological replicates ± SD. (B) Each compound’s target, IC50, CC50, and SI is listed for each virus. (C) Caco-2 cells were pre-treated with the indicated entry inhibitors or Remdesivir positive control in 8-point dose-response for one hour prior to SARS-CoV-2 (MOI 0.5) infection for 48 hours. Cells were processed for automated microscopy and image analysis quantifying total cell numbers (green) (nuclei+) and percent infection (blue) (Spike + /nuclei+) or (GFP + /nuclei+) to determine Percent of Control (POC) % Positive. Data are presented as mean values of n = 2 independent biological replicates ± SD. (D) A549-ACE2 cells were pre-treated with the indicated entry inhibitors or Remdesivir positive control in 8-point dose-response for one hour prior to SARS-CoV-2 (MOI 0.5) infection for 24 hours. Cells were processed for automated microscopy and image analysis quantifying total cell numbers (green) (nuclei+) and percent infection (blue) (Spike + /nuclei+) or (GFP + /nuclei+) to determine Percent of Control (POC) % Positive. Data are presented as mean values of n = 2 independent biological replicates ± SD. (E) Table displaying IC50, CC50, and SI values of the indicated entry inhibitors for Caco-2 and A549-ACE2 cells against SARS-CoV-2. Cells were fixed with 4% formaldehyde and processed for imaging to determine Percent of Control (POC) % Positive for SARS-CoV-2 infection (blue) and total cells (green). Error bars represent the standard deviation of triplicate data for each drug concentration tested in independent experiments.
(TIF)

S3 Fig Variant Sensitivity to Entry Inhibitors is Similar to the Ancestral Strain. (A) Calu-3 cells were pre-treated with the indicated compounds at the indicated concentrations and infected with SARS-CoV-2 variants (MOI 0.2) for 48 hours. Cell lysates were processed for RT-qPCR analysis of viral RNA (Nucleocapsid). (B) A549-ACE2 cells were pre-treated with the indicated compounds at the indicated concentrations and infected with SARS-CoV-2 variants (MOI 0.2) for 24 hours. Cell lysates were processed for RT-qPCR analysis of viral RNA (Nucleocapsid). (C) Huh7.5 cells were pre-treated with the indicated compounds at the indicated concentrations and infected with SARS-CoV-2 variants (MOI 0.2) for 24 hours. Cell lysates were processed for RT-qPCR analysis of viral RNA (Nucleocapsid). Means + SD for individual biological replicates are shown. Significance was calculated using One Way ANOVA with Dunnett Correction on DMSO control (*p < 0.05, **p < 0.01, ***p < 0.001, ****p < 0.0001; ns, no significance.
(TIF)

S4 Fig Staurosporine and Retro-2.1 Block TMPRSS2-dependent Entry Across SARS-CoV-2 Variants. (A) Calu-3 cells were pre-treated with the indicated compounds in 8-point dose-response and infected with SARS-CoV-2 variants (MOI 0.5) for 48 hours followed by automated microscopy and image analysis quantifying total cell numbers (green) (nuclei+) and percent infection (blue) (Spike+ or dsRNA + /nuclei+). Data are presented as mean values of n = 2 independent biological replicates ± SD. (B) Each compound’s IC50, CC50, and SI is listed for each virus.
(TIF)

S5 Fig Staurosporine and Retro-2.1 do not Impact ACE2 Levels. (A) Calu-3 cells were treated with vehicle (DMSO) or indicated compounds at the indicated concentrations for 24 hours. Cell lysates were processed for RT-qPCR analysis of ACE2 or (B) TMPRSS2 mRNA normalized to 18S control. (C) Calu-3, Caco-2, A549-ACE2, and Huh7.5 cells were probed for TMPRSS2 mRNA expression relative to Calu-3. (D) Table listing CT and Fold Change values for TMPRSS2 mRNA expression across cell types relative to Calu-3. (E) Calu-3 cells were treated with vehicle (DMSO) or the indicated compounds at the indicated concentrations for 24 hours. Cell lysates were processed for immunoblot analysis of ACE2 and Tubulin with (F) quantification of relative ACE2 band intensities. (G) A549-ACE2 cells were treated with vehicle (DMSO) or the indicated compounds at the indicated concentrations for 24 hours. Cell lysates were processed for immunoblot analysis of ACE2 and Tubulin with (H) quantification of relative ACE2 band intensities. Means + SD for individual biological replicates are shown. Significance was calculated using One Way ANOVA with Dunnett Correction on DMSO control (*p < 0.05, **p < 0.01, ***p < 0.001; ns, no significance). Representative blots are shown for n = 3.
(TIF)

S6 Fig Additional PKC Inhibitors Do Not Block SARS-CoV-2 Infection. (A) Calu-3 cells were pre-treated with additional PKC inhibitors in 8-point dose-response and infected with SARS-CoV-2 (MOI 0.5) for 48 hours followed by automated microscopy and image analysis quantifying total cell numbers (green) (nuclei+) and percent infection (blue) (Spike+ or dsRNA + /nuclei+). Cytotoxicity was measured using ATPlite (black) following 72-hour treatments. Data are presented as mean values of n = 2 independent biological replicates ± SD. (B) Each compound’s IC50, CC50, and SI is listed for SARS-CoV-2 WA1 in Calu-3 cells.
(TIF)

S7 Fig PKCη is Proviral During SARS-CoV-2 Entry. (A) Calu-3 cells were plated, and RNA was extracted 48 hours later for RNA-seq analysis of PKC isozyme global transcript levels. PKCα, δ, ε, γ, η, ι, and ζ had read counts above 20, whereas PKCθ and PKCβ contained read counts below 20. (B) Calu-3 cells were transfected with pooled or individual siRNAs targeting ACE2, TMPRSS2, PKCγ, or PKCη and at 48 hours infected with SARS-CoV-2 (MOI 0.2) for 48 hours. Cell lysates were processed for RT-qPCR analysis of viral RNA (Nucleocapsid). (C) Calu-3 cells were transfected with pooled or individual siRNAs targeting ACE2, TMPRSS2, or PKCγ and at 48 hours infected with SARS-CoV-2 (MOI 0.2) for 48 hours. Cell lysates were processed for RT-qPCR analysis of PKCγ expression. (D) Calu-3 cells were transfected with pooled or individual siRNAs targeting ACE2, TMPRSS2, or PKCη and at 48 hours infected with SARS-CoV-2 (MOI 0.2) for 48 hours. Cell lysates were processed for RT-qPCR analysis of PKCη expression. (E) Calu-3 cells were transfected with the indicated pooled or individual siRNAs for 48 hours and cell lysates were probed for expression by immunoblot with the indicated antibodies. Representative blots are shown for n = 3. (F) Calu-3 cells were transfected with the indicated siRNAs and processed at 48 hours for automated microscopy and image analysis quantifying total cell numbers (nuclei+) for cell viability. (G) Calu-3 cells were transfected with the indicated siRNA and infected at 48 hours with SARS-CoV-2 Omicron BA.1 (MOI 0.2) for 48 hours prior to processing for automated microscopy and image analysis quantifying total cell numbers (nuclei+) and percent infection (Nucleocapsid + /nuclei+). (H) Calu-3 cells were transfected with the indicated siRNA and infected at 48 hours with SARS-CoV-2 Omicron BA.1 (MOI 0.2) for 48 hours prior to processing for RT-qPCR analysis of viral RNA (Nucleocapsid). (I) Calu-3 cells were transfected with the indicated siRNA and infected at 48 hours with SARS-CoV-2 Omicron BA.5 (MOI 0.2) for 48 hours prior to processing for automated microscopy and image analysis quantifying total cell numbers (nuclei+) and percent infection (Nucleocapsid + /nuclei+). (J) Calu-3 cells were transfected with the indicated siRNA and infected at 48 hours with SARS-CoV-2 Omicron BA.5 (MOI 0.2) for 48 hours prior to processing for RT-qPCR analysis of viral RNA (Nucleocapsid). (K) Calu-3 cells were transfected with non-targeting siRNA control or an siRNA pool targeting PKCη for 48 hours. Cells were treated with 4-fold dilutions of Staurosporine one hour prior to infection with SARS-CoV-2 (MOI 0.2) and fixed for automated microscopy imaging. Infections were normalized to DMSO controls for both non-targeting and PKCη siRNA conditions and expressed as percentage of control (POC). IC_50s_ are displayed with non-linear regression curves from three independent biological replicates with error bars representing the standard error of the mean for each drug concentration tested. (L) Calu-3 cells were transfected with the indicated siRNA and infected at 48 hours with MERS-CoV (MOI 0.2) for 24 hours prior to processing for RT-qPCR analysis of viral RNA (Nucleocapsid). Means + SD for individual biological replicates are shown. Significance was calculated using One Way ANOVA with Dunnett Correction on non-targeting siRNA control (*p < 0.05, **p < 0.01, ***p < 0.001, ****p < 0.0001; ns, no significance.
(TIF)

S8 Fig Retro-2.1 SAR Studies. Calu-3 cells were pre-treated with Retro-2.1 derivatives from MMV in 8-point dose response and infected with SARS-CoV-2 (48 hours; MOI 0.5), VSV-G (24 hours; MOI 0.05), or VSV-S (24 hours; MOI 5) followed by automated microscopy and image analysis quantifying total cell numbers (green) (nuclei+) and percent infection (blue) (Spike+ or dsRNA + /nuclei). Data are presented as mean values of n = 2 independent biological replicates ± SD. Each compound’s IC50, CC50, and SI is listed for each virus in S3 Table.
(TIF)

S9 Fig NF1 is a cellular host factor required for SARS-CoV-2. (A) Calu-3 cells were transfected with the indicated siRNA and processed at 48 hours by automated microscopy and image analysis quantifying total cell numbers (nuclei+) for cell viability. (B) Calu-3 cells were transfected with the indicated siRNA and infected at 48 hours with SARS-CoV-2 Omicron BA.1 (MOI 0.2) for 48 hours prior to processing for automated microscopy and image analysis quantifying total cell numbers (nuclei+) and percent infection (Spike + /nuclei+). (C) Calu-3 cells were transfected with the indicated siRNA and infected at 48 hours with SARS-CoV-2 Omicron BA.1 (MOI 0.2) for 48 hours prior to processing for RT-qPCR analysis of viral RNA (Nucleocapsid). (D) Calu-3 cells were transfected with the indicated siRNA and infected at 48 hours with SARS-CoV-2 Omicron BA.5 (MOI 0.2) for 48 hours prior to processing for automated microscopy and image analysis quantifying total cell numbers (nuclei+) and percent infection (Spike + /nuclei+). (E) Calu-3 cells were transfected with the indicated siRNA and infected at 48 hours with SARS-CoV-2 Omicron BA.5 (MOI 0.2) for 48 hours prior to processing for RT-qPCR analysis of viral RNA (Nucleocapsid). (F) Quantification of relative NF1 immunoblot band intensities in Calu-3 cells following transfections with the indicated siRNAs. (G) Calu-3 cells were transfected with the indicated pooled or individual siRNAs for 48 hours and cell lysates were probed for expression by immunoblot with the indicated antibodies. Representative blots are shown for n = 3. (H) Calu-3 cells were pre-treated with PBS control or 100ng/mL EGF for 20 minutes to induce Ras signaling and infected with SARS-CoV-2 (MOI 0.2) for 48 hours. Cell lysates were processed for RT-qPCR analysis of viral RNA (Nucleocapsid) with significance calculated using unpaired t test with Welch Correction. (I) A549-ACE2 cells were transfected with the indicated siRNA and infected at 48 hours with SARS-CoV-2 (MOI 2) for 24 hours. Cell lysates were processed for RT-qPCR analysis of viral RNA (Nucleocapsid). Means + SD with individual biological replicates are shown. Significance was calculated using One Way ANOVA with Dunnett Correction on non-targeting siRNA control (*p < 0.05, **p < 0.01, ***p < 0.001, ****p < 0.0001; ns, no significance.
(TIF)

S1 Table Primary Screen of Pandemic Response Box. Primary screen data of MMV Pandemic Response Box Compounds (400) against SARS-CoV-2. Calu-3 cells were seeded in 384 well plates and pre-treated the following day with DMSO vehicle control, 10uM of Remdesivir positive control, or 10uM of the Pandemic Response Box (400 compounds) for one hour prior to SARS-CoV-2 (MOI 0.5) infection. After 48 hours, cells were fixed with 4% formaldehyde and processed for imaging to determine Percent of Control (POC) % Positive for SARS-CoV-2 infection, Total Cells for cell viability, and associated Z scores screened in duplicate (Rep A & Rep B).
(XLSX)

S2 Table Dose-response Studies of Pandemic Box SARS-CoV-2 Antivirals. Calu-3 cells were seeded on 384 well plates and pre-treated the following day with the 16 identified antivirals in 8-point dose-response for one hour prior to infection with SARS-CoV-2 (MOI 0.5), VSV-S (MOI 5), VSV-G (MOI 0.05). The Selectivity Index (SI) was calculated from half maximal cytotoxic concentration (CC50)/ the half maximal inhibitory concentration (IC50). ATPlite cytotoxicity assay was determined after 72-hour compound treatments. Dose-response studies identified 10 compounds with a SI > 10 for SARS-CoV-2 WA1. Of these, only Retro-2.1 had a VSV-G SI = 1 and a VSV-S SI > 10.
(XLSX)

S3 Table Retro-2.1 SAR Studies. Calu-3 cells were pre-treated with Retro-2.1 derivatives supplied by MMV in 8-point dose response and infected with SARS-CoV-2 (48 hours; MOI 0.5), VSV-G (24 hours; MOI 0.05), or VSV-S (24 hours; MOI 5) followed by automated microscopy and image analysis quantifying total cell numbers and percent infection. Each compound’s IC50, CC50, and SI is listed for each virus. Cytotoxicity was measured using ATPlite following 72-hour treatments. Data are presented as mean values of n = 2 independent biological replicates ± SD.
(XLSX)

S4 Table List of Antibodies used in this study. (XLSX)

S5 Table List of qPCR primers used in this study. (XLSX)

S6 Table List of siRNA sequences used in this study. (XLSX)
